# Reduced expression in preterm birth of sFLT-1 and PlGF with a high sFLT-1/PlGF ratio in extracellular vesicles suggests a potential biomarker

**DOI:** 10.3389/fendo.2022.1024587

**Published:** 2022-12-22

**Authors:** Sama Hussein, Weina Ju, Stephanie Pizzella, Michael Flory, Chu Chu, Yong Wang, Nanbert Zhong

**Affiliations:** ^1^ New York State Institute for Basic Research in Developmental Disabilities, Staten Island, NY, United States; ^2^ Department of Obstetrics and Gynecology, School of Medicine, Washington University, St. Louis, MO, United States

**Keywords:** sFlt-1, placental growth factor, biomarker, preterm (birth), PTB, extracellular vehicles (EVs), exosomes (EX)

## Abstract

Preterm birth may have a pathological impact on intrauterine development of the fetal brain, resulting in developmental disabilities. In this study, we examine the expression of soluble Fms-like tyrosine kinase 1 (sFLT-1) and placental growth factor (PlGF), which is one of the vascular endothelial growth factors (VEGFs), as these play a key role in angiogenesis; in particular, we examine their effect on the sFLT-1/PlGF ratio in cases of preterm birth as compared to typical pregnancies. Enzyme-linked immunosorbent assay was performed on samples of maternal-derived plasma and extracellular vesicles-exosomes (EVs-EXs) isolated at the third trimester, consisting of 17 samples from cases of preterm birth and 38 control cases. Our results showed that both sFLT-1 (P=0.0014) and PlGF (P=0.0032) were significantly downregulated in cases of preterm birth compared to controls, while the sFLT-1/PIGF ratio was significantly (P=0.0008) increased in EVs-EXs, but not in maternal plasma. Our results suggest that this reduced expression of sFLT-1 and PlGF with an elevated sFLT-1/PlGF ratio in EVs-EXs may represent a potential biomarker for prediction of PTB.

## Introduction

Preterm birth (PTB) has been defined by the World Health Organization (WHO) as any birth before the completion of 37 weeks of gestation. PTB is classified into two major subtypes: spontaneous preterm birth (sPTB) and indicated preterm birth (iPTB). sPTB is in turn categorized into two distinct clinical scenarios: 1) premature onset of labor (POL), which is characterized by the occurrence of regular contractions with associated cervical change and intact membranes, and 2) preterm premature rupture of membranes (pPROM) (1). iPTB occurs when labor is induced or cesarean section delivery carried out due to maternal or fetal disease (2). PTB is responsible for 75% of perinatal deaths and more than 50% of long-term newborn morbidities, including neurological impairments, blindness, deafness, and chronic lung illness, as well as learning difficulties and psychological, behavioral, and social issues (3, 4).

The placenta regulates the fetal environment by controlling the passage of nutrients and waste materials between the maternal and fetal circulations. Placental abnormalities are the most typical form of complications arising during human pregnancies (5). Many prenatal disorders that result in premature delivery involve placental disturbances, generally categorized as malperfused placenta or inflamed placenta (6). An under-perfused placenta is linked to fetal growth restriction, preterm birth owing to early labor or premature rupture of membranes, premature placental detachment (abruptio placenta), and an increased risk of preeclampsia (7).

Angiogenesis is the formation of new microvessels from larger blood vessels; it is an important aspect of embryogenesis, as vascularization of the placenta is required for adequate transport of nutrition and oxygen to the fetus (5). Placental growth is fastest in the first half of pregnancy, and development of placental vascular branching continues until term (8). From day 21 until the end of the first trimester, villous vasculature increases in terms of number of vessels rather than vessel type. Villous vascular development shifts from branching to non-branching angiogenesis at the 26th week of pregnancy and continues in that form until birth, when mature intermediate villi specializing in gas exchange are developed. Placentation is influenced by oxygen levels, angiogenic growth factor(s), and their natural receptors and antagonists (5).

Placental growth factor (PlGF) is a member of the vascular endothelial growth factor (VEGF) family and is predominantly expressed in the placenta (9). FLT-1, a tyrosine-protein kinase, functions as a cell-surface receptor for VEGFA, VEGFB, and PlGF, and is crucial for the formation of the embryonic vasculature, the control of angiogenesis, and other processes (10). FLT-1 (sFLT-1) and PlGF are each expressed differently in the human placenta during pregnancy. Soluble FLT-1 (sFLT-1) is a protein that inhibits angiogenesis; it acts by adhering to the receptor-binding domains of PlGF and VEGF and blocking their interaction on the cell surface, leading to endothelial dysfunction (11). Correlations can be observed between the impact of these growth factors, along with their patterns of expression throughout a pregnancy, and the development of the villous angioarchitecture. sFLT-1 is necessary for embryonic vascular architecture but not for endothelial cell differentiation (12). PlGF binding to sFLT-1 is more likely to occur in the final trimester and to result in non-branching angiogenesis (9).

Extracellular vesicles (EVs), which include exosomes (EXs), are membranous nanovesicles of endocytic origin, measuring 30-150 nm in diameter, that are generated by most cell types in various organisms. They encapsulate various proteins and nucleic acids (microRNA, messenger RNA, long non-coding RNA, and DNA) and are released into the extracellular space, where they circulate. Endosome-specific tetraspanins, including CD9, CD63, and CD81, are abundant in EV-EX membranes (13, 14). Plasma concentrations of EVs-EXs have been found to be more than 50 times higher in pregnant women compared to non-pregnant women (15). EVs-EXs derived from the placenta have been shown to enter the maternal blood in both healthy and pathologic pregnancies, and their concentration rises more than twofold as the pregnancy proceeds, reaching a peak at term. The quantification of placental EVs-EXs in maternal plasma represents fetal growth and might be a valuable biomarker of placental function (15–17).

In this study, we examine the expression of soluble Fms-like tyrosine kinase 1 (sFLT-1) and placental growth factor (PlGF) in EVs-EXs isolated from maternal plasma, in PTB as compared to typical control (Ctrl) pregnancies, as PTB is considered to be a placental disease.

## Material and methods

### Maternal-derived plasma collection:

Plasma samples were obtained from the Department of Obstetrics and Gynecology, Washington University in St. Louis. In total, samples of maternal-derived plasma were collected at the third trimester in 17 cases of PTB, including 8 cases of spontaneous PTB (sPTB) and 9 cases of indicated PTB (iPTB), and 38 typical full-term pregnancies (control); samples were then stored at -80°C until use (**Table 1**). This study was reviewed and approved by the Institutional Review Board (IRB approval #201707152) of Washington University in St. Louis.

**Table 1:** Number of cases and gestational week of preterm births *vs*. controls.

Sample	Number of Cases	Average Gestational Week
**Indicated**	**9**	**32 + 9**
**Spontaneous**	**8**	**35 + 8**
**Control**	**37**	**38 + 8**

### Isolation of EVs-EXs

The System Biosciences EQULTRA-20A-1 ExoQuick Ultra EV isolation kit for serum and plasma was used for EV isolation. We collected 300 μl of plasma and centrifuged at 3,000 × g for 15 minutes to remove cellular debris. 67 μl of ExoQuick was added to 250 μl of supernatant in a fresh tube, which was incubated at 4°C on ice for 30 minutes. Throughout the incubation period, the mixture was mixed well by inverting or flicking the tube. Subsequently, the ExoQuick/plasma mixture was centrifuged at 3,000 g for 10 minutes at room temperature. After centrifugation, the EVs-EXs appeared as a beige or white pellet at the bottom of the tube. The supernatant was carefully aspirated off, and any leftover ExoQuick solution was spun down and completely removed by aspiration. The pellet was then resuspended in 200 μl of Buffer B, and 200 μl of Buffer A was added to the EV-EX mixture. The purification column was centrifuged at 1,000 x g for 30 seconds to remove the storage buffer. It was then washed by adding 500 μl of Buffer B on top of the resin, centrifuging at 1,000 x g for 30 seconds, and then discarding the flow. This step in the process was repeated once. The resin was prepared for sample loading by adding 100 μl of Buffer B on top of it. Finally, the EV-EX mixture was added, and the column was mixed at room temperature on a rotating shaker for 5 minutes. Purified EV-EX was obtained after centrifugation at 1,000 x g for 30 seconds.

### Quantification and normalization of placental EVs-EXs

CD9, a membrane-specific marker of EVs-EXs, was used to represent the internal level of gene expression in EVs-EXs; this was employed for normalization of the expression of PlGF and sFLT-1 in EVs-EXs.

### ELISA

A dilution of the plasma/EVs-EXs (2μl in 4 ml PBS, pH 7.4) was adjusted to the optical density (OD280) indicating 0.04 ng/μl using a nanodrop spectrophotometer. Polystyrene 96-well plates were coated with 50 μl of diluted plasma in PBS and were left at room temperature for one hour on a laboratory rocker; this was followed by incubation overnight at 4°C. Excess plasma was removed from the coated plates; subsequently, 250 μl of blocking buffer (5% milk in PBS) was added and the plates were incubated overnight in a fridge at 4°C. After the incubation period, the blocking buffer was removed and 50 μl per well of primary antisera diluted in PBST (PBS + 0.05% Tween-20) was added in ratios of 1:500, 1:200, and 1:40,000 for anti-CD9 antibody (Monoclonal Santa Cruz sc-13118), PlGF polyclonal antibody (Invitrogen cat #PA5-95604), and sFLT-1 polyclonal antibody (Bioss BS cat #20692R), respectively. The plates were incubated overnight at 4°C on a laboratory rocker. The plates were then washed three times using 300 μl per well of PBST (washing buffer: PBS + 0.05% Tween-20). The following steps were performed in darkness: secondary antisera were diluted in PBST at 1:1000 and 50 μl per well was added. Goat anti-mouse IgG (H+L) secondary antibody, HRP conjugate (Invitrogen cat#31430), was added to CD9 wells, while goat anti-rabbit IgG (H+L) secondary antibody, HRP conjugate (Invitrogen, cat#31460), was used for PlGF and sFLT-1 wells; the plates were covered with aluminum foil and incubated at 37°C for one hour. Next, the plates were washed four times using 300 μl per well of PBST and incubated with 100 μl per well of Thermo Scientific™ Pierce™ TMB Substrate Kit (Thermo Scientific, cat #PI34021; 1:1 peroxide solution and peroxidase substrate) at RT for 30 min on a laboratory rocker, covered with aluminum foil. The reaction was halted by the addition of 100 μl per well of 20% H2SO4, and the absorbance was measured on a microplate reader SpectraMax M3 at 450 nm. Finally, the sFLT-1/PlGF ratio for each sample was determined.

### Statistical analysis

Each sample was measured in duplicate and the average of the two measurements was computed. We computed p-values using the mean of PTB samples against those from typical Ctrl pregnancies. The data collected were tallied, sorted, and statistically evaluated; the normality of the distributions of the optical densities was examined using the Shapiro–Wilk test. All variables were significantly non-normal, with a pronounced rightward skew. Variables were therefore square root transformed, achieving reasonably normal distributions. Differences between groups were analyzed using unpaired two-tailed t-tests, with no adjustments needed for unequal variances. Analyses were performed using version 16.0 of the Stata statistical package (StataCorp, 2019: Stata Statistical Software: Release 16; College Station, TX: StataCorp LLC).

## Results

**Figure 1:**
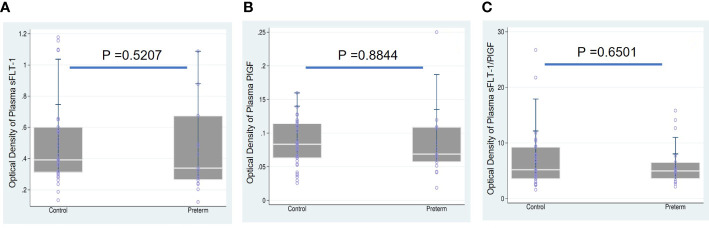
Boxplots overlaid with scatterplots showing median sFLT-1, PlGF, and sFLT1/PlGF ratio in maternal plasma, comparing preterm birth (PTB) and controls (Ctrl). **(A)** sFLT-1 in PTB (0.3385) *vs.* Ctrl (0.3918), **(B)** PlGF in PTB (0.0688) *vs.* Ctrl (0.0833), and **(C)** sFLT-1/PlGF ratio in PTB (4.9779) *vs.* Ctrl (5.2004) showed that sFLT-1, PIGF and sFLT-1/PlGF were not significant in compared to Ctrl groups.

**Figure 2:**
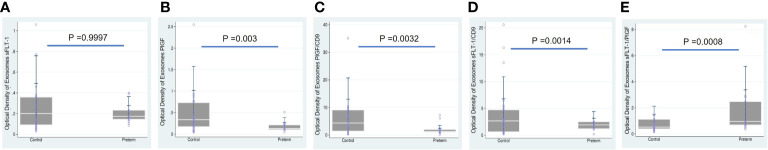
Boxplots overlaid with scatterplots showing median sFLT-1 and PlGF with/without normalization to CD9, and sFLT1/PlGF ratio, each in EVs-EXs, comparing preterm birth (PTB) and controls (Ctrl). **(A)** sFLT-1 in PTB (0.1733) *vs.* Ctrl (0.2001), **(B)** PlGF in PTB (0.1292) *vs.* Ctrl (0.3345), **(C)** PlGF/CD9 in PTB (1.2857) *vs.* Ctrl (4.3520), **(D)** sFLT-1/CD9 in PTB (1.9224) *vs.* Ctrl (2.6671), and **(E)** sFLT-1/PlGF ratio in PTB (0.9578) *vs.* Ctrl (0.5226). As illustrated, sFLT-1 and PlGF are significantly lower in PTB (p<0.05), while the sFLT-1/PlGF ratio is significantly higher (p<0.05) in PTB compared to the Ctrl group.

We first tested whether women who gave birth to a preterm fetus had altered levels of sFLT-1 and PlGF, as measured with plasma and EVs-EXs. As shown in **Table 2**, individual measurement of sFLT-1 and PlGF indicated that there was little difference in the level of either sFLT-1 or PlGF in PTB samples as compared to controls, showing no statistical significance with P > 0.05. There was also no significant difference in terms of the sFLT-1/PlGF ratio in plasma (**Figure 1**). Our immediate hypothesis was that this non-significance was a result of heterogenous sampling in our study: i.e., among 17 PTB samples, nine were from cases of iPTB and eight were from cases of sPTB, which could interfere with the results. To verify this, we analyzed our data by separating iPTB from sPTB samples and repeating the statistical analysis. Surprisingly, there was no significant effect for sPTB or iPTB in three comparisons (namely, sPTB *vs*. Ctrl, iPTB *vs*. Ctrl, and PTB *vs*. Ctrl; P > 0.05, data not shown). Because there were no statistical differences in the comparisons of sPTB *vs*. iPTB *vs*. PTB, we decided to combine sPTB with iPTB, treating them as a single group for subsequent analyses. Considering the fact that both sFLT-1 and PlGF are involved in placental development, measurement of placental sFLT-1 and PlGF would provide a more accurate reflection of their expression in pregnancy. We therefore reanalyzed sFLT-1 and PlGF levels, and the sFLT-1/PlGF ratio, with EVs-EXs that were isolated from maternal plasma but released from trophoblasts (15, 16). Indeed, the results of this analysis showed that there was a striking difference between the sample groups in sFLT-1, PlGF, and sFLT-1/PlGF ratio within EVs-EXs, when CD9 was used to normalize the assay (**Figure 2**). The corresponding P values were 0.0014 for sFLT-1/CD9, 0.0032 for PlGF/CD9, and 0.0008 for sFLT-1/PlGF. If CD9 was not used for normalization, PIGF level was still found to be significantly different, with P = 0.0003, although this was not the case for sFLT-1 level, with P > 0.05.

**Table 2:** Quantitative measurement of sFLT-1 and PlGF.

Sample Type	Biomarker	Median in Preterm	Median in Control	P-Value	Significance
**EVs-EXs**	**PlGF**	**0.1292**	**0.3345**	**0.0003**	**Significant**
**EVs-EXs**	**PlGF/CD9**	**1.2857**	**4.3520**	**0.0032**	**Significant**
**EVs-EXs**	**sFLT-1**	**0.1733**	**0.2001**	**0.9997**	**Non-Significant**
**EVs-EXs**	**sFLT-1/CD9**	**1.9224**	**2.6671**	**0.0014**	**Significant**
**EVs-EXs**	**sFLT-1/PlGF ratio**	**0.9578**	**0.5226**	**0.0008**	**Significant**
**Maternal plasma**	**PlGF**	**0.0688**	**0.0833**	**0.8844**	**Non-Significant**
**Maternal plasma**	**sFLT-1**	**0.3385**	**0.3918**	**0.5207**	**Non-Significant**
**Maternal plasma**	**sFLT-1/PlGF ratio**	**4.9779**	**5.2004**	**0.6501**	**Non-Significant**

## Discussion

The primary underlying mechanism in numerous pregnancy complications is now understood to be disorders of placentation (18). Many factors influence the health of the placenta, including ethnicity, history of smoking cigarettes, high blood pressure, multiple gestation pregnancy, maternal blood-clotting disorders, history of uterine surgery (such as a cesarean delivery), history of placental problems, maternal substance abuse (such as cocaine use), abdominal trauma (such as from a fall or blunt trauma), maternal age (as women over the age of 40 years have a higher risk of developing placental problems), and premature rupture of membranes (because the risk of placental problems increases when the amniotic sac ruptures too early). Phenotypically, these factors may result in adverse pregnancy outcomes, such as miscarriage or stillbirth, IUGR, preeclampsia, or spontaneous preterm birth, which are considered to be placental diseases. Pathogenically, these diseases may share abnormal development and differentiation of the placenta at an early stage of placentation, which may impact placental angiogenesis. Molecularly, altered placental angiogenesis, under the influence of the risk factors mentioned above, may result from differential gene expression of angiogenic factors, causing these angiogenic factors to be up- or down-regulated in the placenta, which would also be reflected in maternal circulation. Therefore, alteration of the pro- and anti-angiogenic proteins that have been identified and characterized in relation to each individual disorder could be applied as a biomarker that can be clinically used to predict pregnancy disorder(s), such as preeclampsia or miscarriage.

In preeclampsia (PE) it has been well established that increased levels of sFLT-1 and reduced levels of PlGF may be the underlying pathophysiology (19). The sFLT-1/PlGF ratio has been demonstrated to be elevated in pregnant women 4-5 weeks before the clinical onset of preeclampsia (20). In intrauterine growth retardation (IUGR), few studies have investigated maternal serum levels of sFLT-1 and PlGF. According to one report, the median level of sFLT-1 is significantly greater in women who experience PE and IUGR compared to controls, whereas the median level of PlGF is lower (21). Additionally, a previous study on mice models of fetal growth retardation has shown that elevated sFLT-1 levels disrupt vascularization in the murine placenta, impair placental function, and result in fetuses with fetal growth retardation (22). Finally, in relation to miscarriage, it has been reported that concentrations of sFLT-1 and PlGF are significantly lower in a subgroup of participants with threatened miscarriage who subsequently experience miscarriage, compared to asymptomatic controls (10); the same has been observed in cases of ectopic pregnancy or missed abortion, compared with healthy intrauterine pregnancies (23).

Previously, we have found that cytokine–cytokine receptor interaction is the most common and the most enriched pathway observed in spontaneous preterm birth and spontaneous miscarriage. Ten genes (CCL3, TNF, CCL2, CXCL3, TNFRSF8, CCL4, CXCL10, CXCR4, CCL3L3, and CCL4L1) that are commonly differentially expressed in both sPTB and spontaneous miscarriage (sM) are largely focused on chemokines (including the CC subfamily and the CXC subfamily), TNF, and TNFRSF8, suggesting that sPTB and sM may share a common pathogenic mechanism. Considering that preterm birth is one of the placental diseases that has been determined to share pathogenic alteration in the early stage of placentation with miscarriage and preeclampsia (17, 24), we measured sFLT-1 and PlGF in our preterm birth cohort. Indeed, our results demonstrated that sFLT-1 and PlGF are differentially expressed in PTB.

CD9, CD63, and CD81 are membrane proteins that have become widely accepted as extracellular vesicle markers. CD63 and CD81 have been previously employed as quantifiers of total extracellular vesicle particles, including exosomes, in circulation in maternal blood (25–27). In this study, CD9 was employed as an internal reference for EVs-EXs; without normalization against CD9, the sFlT-1 findings were not significant (**Figure 2A**). This aspect of the findings highlights the importance of using CD9 and supports the hypothesis that, without normalization, sFLT-1 expression levels may be derived not only from exosomes, but also from other sources. In the present study, we found that PlGF/CD9 was 71% lower in the PTB group compared to the typical pregnancy group, and the same was true of sFLT-1/CD9 (47% lower), while the sFLT-1/PlGF ratio was significantly higher at 147% (**Figure 2**). Conversely, when we examined sFLT-1, PlGF, and sFLT-1/PlGF in plasma, the findings were not significant (**Figure 1**). This indicates that placental-derived EVs-EXs in maternal plasma are the better materials for studying placental function and intrauterine fetal growth. This result also opened a new avenue for the application of quantitative measurement of sFLT-1, PlGF, and sFLT-1/PlGF in EVs-EXs as a potential biomarker for prediction of sPTB.

In summary, PlGF is commonly reduced in placental diseases, such as PE, IUGR, miscarriage, and preterm birth. While the sFLT-1/PlGF ratio is also commonly elevated, which could arise as a result of the reduced levels of PlGF, use of the sFLT-1/PlGF ratio alone as a biomarker would be misleading. However, a combination of individual sFLT-1 or PlGF assay with the sFLT-1/PlGF ratio could be more accurate. This combination may also differentiate sPTB from PE and from IUGR. To further confirm the potential value of employing sFLT-1, PlGF, and sFLT-1/PlGF as a set of biomarkers for prediction of sPTB, further studies should be conducted with a cohort offering a larger sample size at early-stage pregnancy. For this purpose, employing EVs-EXs rather than plasma is highly recommended, not only for the prediction of sPTB but also for intrauterine loss of pregnancy, IUGR, or PE.

## Data availability statement

The raw data supporting the conclusions of this article will be made available by the authors, without undue reservation.

## Ethics statement

The studies involving human participants were reviewed and approved by Yong Wang Department of Obstetrics and Gynecology School of Medicine, Washington University IRB 201707152. The patients/participants provided their written informed consent to participate in this study.

## Author contributions

NZ conceived, designed, and supervised the project study. SH designed experiments and performed experiments with WJ and CC. YW and SP provided the samples. SH and MF performed statistical analysis. SH drafted, and NZ finalized, the manuscript. All authors read and approved the final manuscript.

## References

[1] Ozer E. Placenta in preterm birth. Erez O, editor. London: Preterm Birth, IntechOpen (2013). doi: 10.5772/54887

[2] Stout MJ Busam R Macones GA Tuuli MG. Spontaneous and indicated preterm birth subtypes: interobserver agreement and accuracy of classification. Am J Obstet Gynecol. (2014) 211(5):530.e1–4. doi: 10.1016/j.ajog.2014.05.023 PMC4234690 24844852

[3] Colvin M McGuire W Fowlie PW. Neurodevelopmental outcomes after preterm birth. BMJ (2004) 329(7479):1390–3. doi: 10.1136/bmj.329.7479.1390 PMC535458 15591566

[4] Faye-Petersen OM. The placenta in preterm birth. J Clin Pathol (2008) 61:1261–75. doi: 10.1136/jcp.2008.055244 19074631

[5] Barut F Barut A Gun BD Kandemir NO Harma MI Harma M. Intrauterine growth restriction and placental angiogenesis. Diagn Pathol (2010) 5:24. doi: 10.1186/1746-1596-5-24 20412591 PMC2865442

[6] Garfinkle J Miller S. The placenta and neurodevelopment in preterm newborns. NeoReviews (2018) 19:e456–66. doi: 10.1542/neo.19-8-e456

[7] Catov JM Muldoon MF Reis SE Ness RB Nguyen LN Yamal JM. Preterm birth with placental evidence of malperfusion is associated with cardiovascular risk factors after pregnancy: a prospective cohort study. BJOG (2018) 125(8):1009–17. doi: 10.1111/1471-0528.15040 PMC6013820 29193660

[8] Lee MY Huang JP Chen YY Aplin JD Wu YH Chen CY. Angiogenesis in differentiated placental multipotent mesenchymal stromal cells is dependent on integrin alpha5beta1. PloS One (2009) 4(10):e6913. doi: 10.1371/journal.pone.0006913 19847290 PMC2760707

[9] Wu WB Xu YY Cheng WW Yuan B Zhao JR Wang YL. Decreased PGF may contribute to trophoblast dysfunction in fetal growth restriction. Reproduction (2017) 154(3):319–29. doi: 10.1530/REP-17-0253 28676532

[10] Muttukrishna S Swer M Suri S Jamil A Calleja-Agius J Gangooly S. Soluble flt-1 and PlGF: new markers of early pregnancy loss? PloS One (2011) 6(3):e18041. doi: 10.1371/journal.pone.0018041 21448460 PMC3063178

[11] Miyake T Kumasawa K Sato N Takiuchi T Nakamura H Kimura T. Soluble VEGF receptor 1 (sFLT1) induces non-apoptotic death in ovarian and colorectal cancer cells. Sci Rep (2016) 6:24853. doi: 10.1038/srep24853 27103202 PMC4840331

[12] Fong GH Rossant J Gertsenstein M Breitman ML. Role of the flt-1 receptor tyrosine kinase in regulating the assembly of vascular endothelium. Nature (1995) 376(6535):66–70. doi: 10.1038/376066a0 7596436

[13] Czernek L Düchler M. Exosomes as messengers between mother and fetus in pregnancy. Int J Mol Sci (2020) 21(12):4264. doi: 10.3390/ijms21124264 32549407 PMC7352303

[14] Khushman M Bhardwaj A Patel GK Laurini JA Roveda K Tan MC. Exosomal markers (CD63 and CD9) expression pattern using immunohistochemistry in resected malignant and nonmalignant pancreatic specimens. Pancreas (2017) 46(6):782–8. doi: 10.1097/MPA.0000000000000847 PMC5494969 28609367

[15] Salomon C Torres MJ Kobayashi M Scholz-Romero K Sobrevia L Dobierzewska A. A gestational profile of placental exosomes in maternal plasma and their effects on endothelial cell migration. PloS One (2014) 9(6):e98667. doi: 10.1371/journal.pone.0098667 24905832 PMC4048215

[16] Ghafourian M Mahdavi R Akbari Jonoush Z Sadeghi M Ghadiri N Farzaneh M. The implications of exosomes in pregnancy: emerging as new diagnostic markers and therapeutics targets. Cell Commun Signal (2022) 20(1):51. doi: 10.1186/s12964-022-00853-z 35414084 PMC9004059

[17] Fang Y Wan C Wen Y Wu Z Pan J Zhong M. Autism-associated synaptic vesicle transcripts are differentially expressed in maternal plasma exosomes of physiopathologic pregnancies. J Transl Med (2021) 19(1):154. doi: 10.1186/s12967-021-02821-6 33858444 PMC8051067

[18] Brosens I Pijnenborg R Vercruysse L Romero R. The “Great obstetrical syndromes” are associated with disorders of deep placentation. Am J Obstet Gynecol. (2011) 204(3):193–201. doi: 10.1016/j.ajog.2010.08.009 21094932 PMC3369813

[19] Levine RJ Maynard SE Qian C Lim KH England LJ Yu KF. Circulating angiogenic factors and the risk of preeclampsia. N Engl J Med (2004) 350(7):672–83. doi: 10.1056/NEJMoa031884 14764923

[20] Ohkuchi A Saito S Yamamoto T Minakami H Masuyama H Kumasawa K. Short-term prediction of preeclampsia using the sFlt-1/PlGF ratio: a subanalysis of pregnant Japanese women from the PROGNOSIS Asia study. Hypertension Res (2021) 44(7):813–21. doi: 10.1038/s41440-021-00629-x PMC8255209 33727707

[21] Birdir C Droste L Fox L Frank M Fryze J Enekwe A. Predictive value of sFlt-1, PlGF, sFlt-1/PlGF ratio and PAPP-a for late-onset preeclampsia and IUGR between 32 and 37 weeks of pregnancy. Pregnancy Hypertens (2018) 12:124–8. doi: 10.1016/j.preghy.2018.04.010 29674192

[22] Vogtmann R Kühnel E Dicke N Verkaik-Schakel RN Plösch T Schorle H. Human sFLT1 leads to severe changes in placental differentiation and vascularization in a transgenic hsFLT1/rtTA FGR mouse model. Front Endocrinol (Lausanne). (2019) 10:165. doi: 10.3389/fendo.2019.00165 30949132 PMC6437783

[23] Daponte A Pournaras S Polyzos NP Tsezou A Skentou H Anastasiadou F. Soluble FMS-like tyrosine kinase-1 (sFlt-1) and serum placental growth factor (PlGF) as biomarkers for ectopic pregnancy and missed abortion. J Clin Endocrinol Metab (2011) 96(9):E1444–51. doi: 10.1210/jc.2011-0037 21715541

[24] Wang P Pan J Tian X Dong X Ju W Wang Y. Transcriptomics-determined chemokine-cytokine pathway presents a common pathogenic mechanism in pregnancy loss and spontaneous preterm birth. Am J Reprod Immunol (2021) 86(1):e13398. doi: 10.1111/aji.13398 33565696 PMC8243792

[25] Elahi FM Casaletto KB Altendahl M Staffaroni AM Fletcher E Filshtein TJ. “Liquid biopsy” of white matter hyperintensity in functionally normal elders. Front Aging Neurosci (2018) 10:343. doi: 10.3389/fnagi.2018.00343 30483114 PMC6244607

[26] Miranda J Paules C Nair S Lai A Palma C Scholz-Romero K. Placental exosomes profile in maternal and fetal circulation in intrauterine growth restriction - liquid biopsies to monitoring fetal growth. Placenta (2018) 64:34–43. doi: 10.1016/j.placenta.2018.02.006 29626979

[27] Sarker S Scholz-Romero K Perez A Illanes SE Mitchell MD Rice GE. Placenta-derived exosomes continuously increase in maternal circulation over the first trimester of pregnancy. J Transl Med (2014) 12:204. doi: 10.1186/1479-5876-12-204 25104112 PMC4283151

